# Poly(Pyridinium Salt)s Containing 2,7-Diamino-9,9′-Dioctylfluorene Moieties with Various Organic Counterions Exhibiting Both Lyotropic Liquid-Crystalline and Light-Emitting Properties

**DOI:** 10.3390/molecules26061560

**Published:** 2021-03-12

**Authors:** Pradip K. Bhowmik, Tae S. Jo, Jung J. Koh, Jongwon Park, Bidyut Biswas, Ronald Carlo G. Principe, Haesook Han, András F. Wacha, Matti Knaapila

**Affiliations:** 1 Department of Chemistry and Biochemistry, University of Nevada Las Vegas, 4505 S. Maryland Parkway Box 454003, Las Vegas, NV 89154-4003, USA; chotaesoo@hotmail.com (T.S.J.); jung.koh@unlv.edu (J.J.K.); jongwonpark8937@gmail.com (J.P.); biswasbidyut@gmail.com (B.B.); princr1@unlv.nevada.edu (R.C.G.P.); hanh3@unlv.nevada.edu (H.H.); 2 Research Centre for Natural Sciences, Institute of Materials and Environmental Chemistry, Magyar Tudósok körútja 2, 1117 Budapest, Hungary; wacha.andras@ttk.hu; 3 Department of Physics, Technical University of Denmark, 2800 Kgs. Lyngby, Denmark; matti.knaapila@fysik.dtu.dk

**Keywords:** poly(pyridinium salt)s, metathesis reaction, gel permeation chromatography, hairy-rod polymers, lyotropic liquid-crystalline phase, polarizing optical microscopy, SAXS, differential scanning calorimetry, thermogravimetric analysis, luminescence, UV-Vis spectroscopy, aggregation

## Abstract

A series of poly(pyridinium salt)s-fluorene main-chain ionic polymers with various organic counterions were synthesized by using ring-transmutation polymerization and metathesis reactions. Their chemical structures were characterized by Fourier Transform Infrared (FTIR), proton (^1^H), and fluorine 19 (^19^F) nuclear magnetic resonance (NMR) spectrometers. These polymers showed a number-average molecular weight (M_n_s) between 96.5 and 107.8 kg/mol and polydispersity index (PDI) in the range of 1.12–1.88. They exhibited fully-grown lyotropic phases in polar protic and aprotic solvents at different critical concentrations. Small-angle X-ray scattering for one polymer example indicates lyotropic structure formation for 60–80% solvent fraction. A lyotropic smectic phase contains 10 nm polymer platelets connected by tie molecules. The structure also incorporates a square packing motif within platelets. Thermal properties of polymers were affected by the size of counterions as determined by differential scanning calorimetry and thermogravimetric analysis measurements. Their ultraviolet-visible (UV-Vis) absorption spectra in different organic solvents were essentially identical, indicating that the closely spaced π-π* transitions occurred in their conjugated polymer structures. In contrast, the emission spectra of polymers exhibited a positive solvatochromism on changing the polarity of solvents. They emitted green lights in both polar and nonpolar organic solvents and showed blue light in the film-states, but their λ_em_ peaks were dependent on the size of the counterions. They formed aggregates in polar aprotic and protic solvents with the addition of water (*v*/*v*, 0–90%), and their λ_em_ peaks were blue shifted.

## 1. Introduction

The phenylated poly(pyridinium salt)s are a class of main-chain ionic polymers, also known as ionene, polymers that are usually prepared using a ring-transmutation polymerization reaction of bispyrylium salts and diamines, and metathesis reactions [1,2,3,4,5]. Depending on the chemical structures of bispyrylium salts and diamines, they can be π-conjugated or non-conjugated ionic polymers. Various π-conjugated ionic polymers exhibit thermotropic liquid-crystalline (LC) and light-emitting properties in both solution and solid-state [6,7,8]; others exhibit both thermotropic and lyotropic phases, also called amphotropic LC properties combined with the light-emitting properties in both solution and solid-state [9,10,11]. Much of this behavior may be understood in terms of supramolecular hairy-rod type polymers [12,13,14]. Additionally, π-conjugated, and even non-conjugated ionic polymers exhibit lyotropic LC phases in both protic and aprotic solvents and light-emitting properties in both solution and solid-state depending on their chemical microstructures [15,16,17,18,19,20,21,22]. They can be appropriately called functional ionic polymers as delineated (vide infra). The dispersion of single-walled carbon nanotubes with poly(pyridinium salt)s via non-covalent interactions in dimethyl sulfoxide can be achieved for the preparation of carbon nanotube-based composites [23,24,25]. Specifically, the tailored poly(pyridinium salt)s with aromatic phosphine oxide moieties in their main-chain chains are found to be excellent inherently fire-retardant ionic polymers [26]. They can be used for the electrochromic materials for the potential application for smart windows since they have redox properties of the main chain ionic moieties [27,28,29]. Even they have touted for the potential use of the nonlinear optical properties using both aromatic and aliphatic moieties in their main chains [30]. Additionally, they can be used for the detection of various types of biomolecules based on their optical properties of phenylated pyridinium and aromatic diamine moieties exhibit [31,32,33,34,35,36]. For example, one such poly(pyridinium salt)s prepared from calix [4], arene diamine, showed an interesting result, *Pseudomonas fluorescens* DNA has a strong interaction with this polymer predominantly by electrostatic interactions for sensing as evaluated by fluorescent titration and transmission electron microscopy studies [31]. Another polymer prepared from benzidine giving rise to a conjugated polyelectrolyte that was used to develop a sensitive fluorescence-based biosensor for homogeneous DNA detection [32]. Even a conjugated poly(pyridinium salt)s based on 3,6-diamino-N-butylcarbazole exhibited aggregation-induced light emission properties. A fluorescence turn-on biosensor for calf thymus DNA detection and quantification has been developed because of this interesting property [33]. Finally, they are suitable components for the construction of multilayer assemblies with various anionic polymers by the sequential layer-by-layer deposition techniques through electrostatic interactions. These techniques have received significant interest globally to create numerous functional materials [37,38,39].

Among all the conjugated polymers studied to date for optoelectronic applications, including polymer light-emitting diodes (PLEDs), field-effect transistors (FETs), and polymer solar cells (PSCs), polyfluorenes (PFs) are the shining stars because of their widespread use in these devices [40,41,42,43,44,45,46,47,48,49]. They are one of the most promising polymers for blue-light emitters because of their high photoluminescence (PL) and electroluminescence (EL) efficiencies and thermal stability. Their color properties can be tuned by introducing low-band gap co-monomers into their polymer backbone or attaching different functional groups onto a fluorene unit. In spite of these advantages, there are some limitations that hamper their potential applications. In general, to obtain highly efficient PLED devices, suppressing the intermolecular interaction such as aggregation between molecules is required. However, undesirable green emission of PFs can be observed upon thermal annealing or device operation. This low-band gap emission can be related to the formation of excimers or aggregation in solid-state, leading to a red-shifted emission. In the PLED application, the balance electron/hole transport through the device is crucial, and it can be achieved by incorporating charge transporting layer(s), or doping charge transporting materials into the emissive layer, or introducing electron withdrawing/donating segments into the polymer repeating units. From the synthetic viewpoint, the introduction of electron and/or hole transporting monomers into polymer main chains is a common and efficient approach employed in polymer chemistry. Additionally, PFs and their copolymers have been gaining increasing interest as conjugated polyelectrolytes both as cationic and anionic groups in side chains because they exhibit relatively high-emission quantum yields in aqueous solutions and emit blue light, and their chemical structures can be easily tuned through the substitution at the C9 fluorene position [50,51,52,53,54,55,56,57,58,59,60,61,62,63,64]. Various protocols have been developed over the decades to detect biomolecules as biosensors. However, many of these protocols are based on the Föster resonance energy transfer mechanism that requires a costly and elaborate process [59,60,61,62,63]. In general, conjugated polyelectrolytes, including PFs are synthesized by transition metal-based cross-coupling reactions. The importance of these coupling reactions cannot be overstated; however, in a practical sense, the presence of residual metal species in final products can limit applications or necessitate elaborate purification procedures. Post-functionalization of fluorenes to introduce ionic groups such as quaternary ammoniums groups and sulfonated groups also remain a challenge. Thus, the development of new conjugated polyelectrolytes (transition metal-free method) in a single-step method for the detection of biomolecules is still required.

**Scheme 1:**
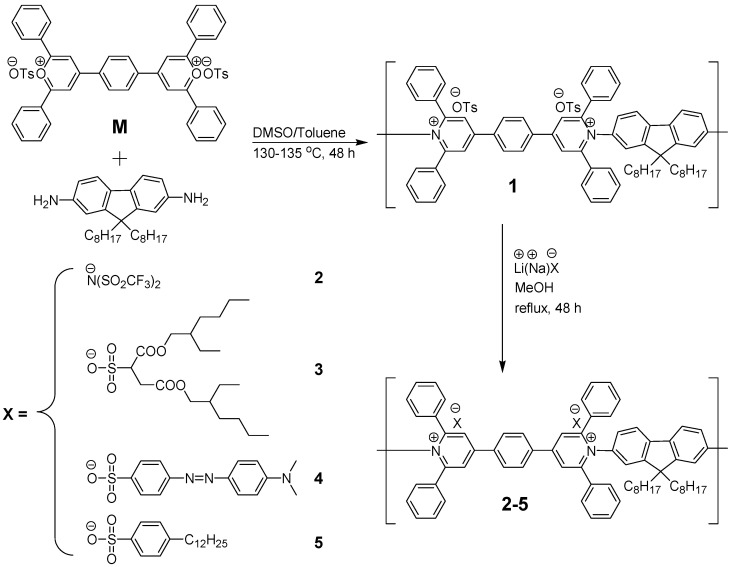
Synthesis of poly(pyridinium salt)s-fluorenes, **1**–**5**.

In this article, we synthesized a series of poly(pyridinium salt)s-fluorenes, **1**–**5**, of main-chain ionic polymers using a ring-transmutation polymerization reaction in dimethyl sulfoxide and a metathesis reaction in methanol (Scheme 1). This polymerization reaction is a transition metal-free method for the preparation of high molecular weight main-chain cationic PFs. Their chemical structures, thermal and optical properties are studied by several experimental techniques. These results show that by changing the polarity of solvents and size of the counterions, the emission color of the ionic polymers, **1**–**5**, could be tuned from blue to green light in solution and solid states.

## 2. Results and Discussion

### 2.1. Chemical Structures of Polymers ***1***–***5***

**Figure 1:**
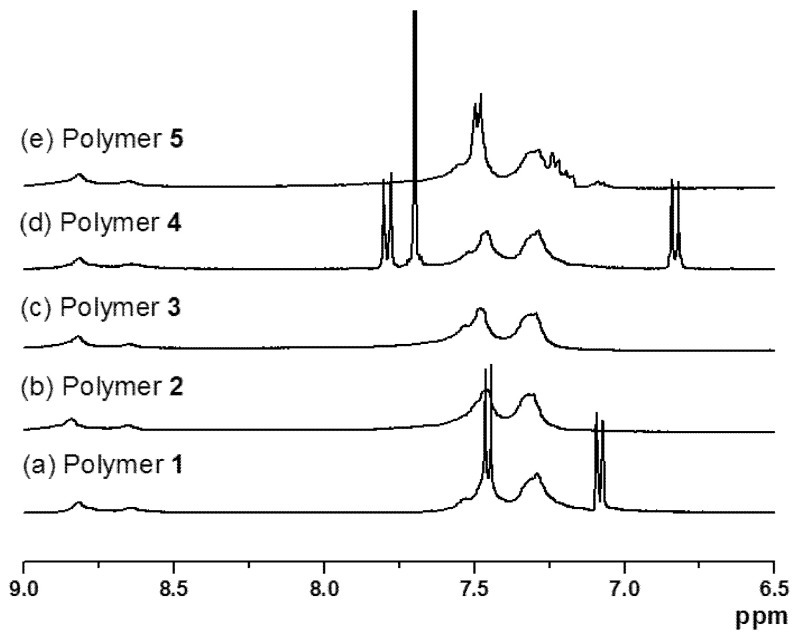
Expanded ^1^H NMR spectra of polymers **1**–**5** (10 mg/mL in *d_6_*-DMSO at 25 °C).

Polymer **1** was synthesized with a reaction between bispyrylium salt, **M**, and 9,9′-dioctyl-9*H*-fluorene-2,7-diamine in dimethyl sulfoxide (DMSO) at 130–135 °C for 48 h under a nitrogen atmosphere [5]. To increase the molecular weight of the polymer, a few mL of toluene was added into the solution to remove generated water during the reaction by a Dean-Stark trap; whereas polymers **2**–**5** were made by a metathesis reaction in methanol by changing the tosylate counterion to various organic counterions as outlined in Scheme 1. Their chemical structures were confirmed by FTIR, ^1^H, and ^19^F and spectroscopy and elemental analyses. The signals of carbon nuclei of polymers could not be obtained even at elevated temperatures because of the viscous solution of these polymers in DMSO. Moreover, the relatively high viscosity of each of these polymers and the broadness of its proton spectrum were suggestive of the high molecular weight of polymers. The FTIR spectra (Figure S1) showed the representative characteristic peaks for polymer **1**: 1620–1448 (C=C and C=N aromatic ring stretching), 1196 (C–N^+^), 1119 (S=O asymmetric stretching), and 1033–1010 (S=O symmetric stretching). After the exchange of tosylate to triflimide, an additional C–F stretching vibration at ν = 1350 cm^−1^ in polymer **2** was observed, indicating the presence of triflimide counterion. Moreover, polymers **3** and **4** displayed unique additional peaks at ν = 1736 cm^−1^ (C=O stretching) from dioctyl sulfosuccinate counterion and 1458 and 1365 cm^−1^ (N=N and C–N stretching) from methyl orange, respectively. The expanded ^1^H NMR spectrum of polymer **1** (Figure 1) showed characteristic broad peaks at δ = 8.82 and 8.62 ppm for the protons of the aromatic moieties of poly(pyridinium salt) and a set of resonances at δ = 7.45, 7.08 and 2.27 ppm for the protons of the aromatic moiety and methyl group in the tosylate counterion. A broad triplet at δ = 0.88 revealed the presence of dioctyl fluorene moiety in the repeating unit (see Supporting Information). Exchange of counterion from tosylate to triflimide was confirmed by the disappearance of tosylate resonances (Figure 1) and appearance of a new signal at δ = −78.32 ppm in ^19^F NMR spectrum (Figure S2), resulting in the completion of the reaction. For polymer **3**, new additional peaks from the dioctyl sulfosuccinate counterion appeared in the range of the aliphatic region, and their ^1^H integral ratio was in good agreement with each other. After changing the counterion from tosylate to methyl orange, a new set of signals was observed at δ = 7.79, 7.70, 6.80 (aromatic protons), and 3.06 (–N(CH_3_)_2_) in the ^1^H NMR spectrum. This result indicated the metathesis reaction underwent completion without causing any side reactions. The polymer **5**, which had dodecylbenzene sulfonate as the counterion, showed the expected proton signals in its NMR spectrum. The full spectra of polymers **1**–**5** are provided in the Supplementary Materials (Figure S3).

### 2.2. Molecular Weights of Polymers ***1***–***5***

**Figure 2:**
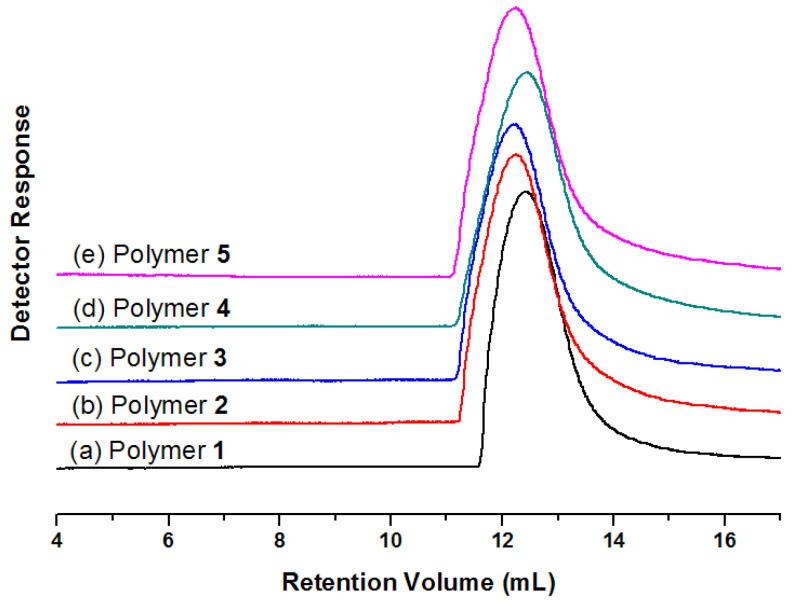
Gel permeation chromatography (GPC) plots (2 mg/mL in DMSO with 0.01 M LiBr) of polymers **1**–**5**.

Molecular weight properties (number-average molecular weights (M_n_) and polydispersity index (PDI)) of the polymers, **1**–**5**, in dimethyl sulfoxide were investigated using gel permeation chromatography (GPC) with the interdetector signals such as refractometer, viscometer, and low- and right-angle light scattering. To minimize the polyelectrolyte interaction between polymers, a small amount of LiBr was used to suppress the ionic interaction between the polymer chains and the interaction with GPC column packing material. The data, including the radius of gyration and hydrodynamic volumes of synthesized polymers, **1**–**5**, are compiled in Table 1 and plotted in Figure 2. For polymers **1**–**5**, the number-average molecular weight (M_n_s) values were in the range of 96.5–107.8 kg/mol, and PDI values were between 1.12 and 1.88. As expected, the molecular weights and PDI values of these polymers were essentially in a similar range since polymer **2**–**5** were synthesized by the metathesis reactions of polymer **1** with the corresponding salts in an organic solvent. It is reasonable to assume that all these ionic polymers had similar molecular weights for a meaningful comparison of their lyotropic liquid-crystalline properties. In other words, thermal, solution, optical, and physical properties can be further studied without concerns for the secondary effects of molecular weights on these polymers.

### 2.3. Solution Properties of ***1***–***5*** by Polarized Optical Microscopy

In general, highly π-conjugated polymers have some limitations in processing them into thin films or fibers due to their poor solubility in common organic solvents. To increase the solubility of the polymers, long alkyl chains or flexible linkages were introduced to enhance the interaction between polymer and solvents. As reported earlier, poly(pyridinium salt)s with various linkages in the main chain or organic counterions showed good solubility in organic solvents while keeping high molecular weights [4,5,6,9,11,16,17].

The synthesized poly(pyridinium salt)s-fluorene, **1**–**5**, displayed good solubility in DMSO, acetonitrile (CH_3_CN), and MeOH, and this information motivated us to investigate lyotropic LC properties of the synthesized polymers. It has been reported that many poly(pyridinium salt)s exhibit the lyotropic LC properties in both aprotic and protic polar solvents at various critical concentrations (C*) depending on the rigidity of backbones of polymer structures [4,5,6,9,11,16,17].

**Figure 3:**
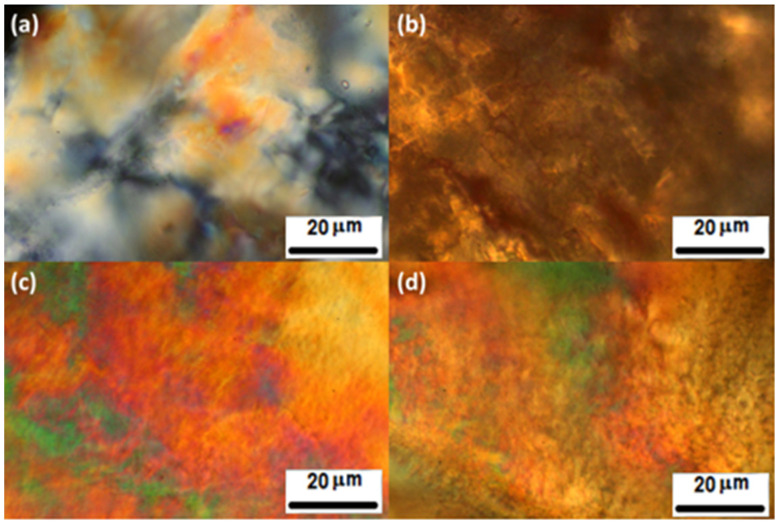
Photomicrographs of (**a**) polymer **1** at 30 wt% in DMSO, (**b**) polymer **2** at 20 wt% in CH_3_CN, (**c**) polymer **3** at 30 wt% in CH_3_CN, and (**d**) polymer **5** at 40 wt% in MeOH under crossed polarizers exhibiting lyotropic liquid-crystalline (LC) phase, respectively (magnification 400×).

Polarized optical microscopy indicates that polymer **1** containing tosylate (OTs) as counterions formed isotropic solution in the range of 1–10 wt%, a biphasic phase at 20 wt%, and a lyotropic phase at 30 wt% in DMSO (Table 2). Furthermore, such lyotropic properties of polymer **1** were observed at 20 wt% in acetonitrile and methanol, respectively. Similarly, polymer **2** with triflimide counterions displayed lyotropic properties at 30 and 20 wt% in DMSO and CH_3_CN, respectively. However, there was no development of the LC phase in CH_3_OH because of its poor solubility resulting in the phase separation of polymer and solvent. In contrast to polymers **1** and **2**, polymer **3** formed a lyotropic LC phase at relatively high concentrations 40 wt% in DMSO and 30 wt% in both CH_3_CN and CH_3_OH, respectively, because of the presence of bulky and long alkyl chain counterions inducing the increased solubility in these solvents. In general, there are several key factors to determine the formation of the lyotropic properties in a polymer, such as rod-like structures with an extended chain character to facilitate the alignment of the polymer chain along a particular direction or sufficient solubility to exceed the critical concentration. The solubility and chain stiffness of a polymer is dependent on the microstructure, molecular weight, polymer-polymer and polymer-solvent interactions, and temperature [65,66,67,68,69,70]. Therefore, the presence of bulky, flexible organic counterions in poly(pyridinium salt)s might increase these interactions resulting in the increased solubility and the prospect of the formation of a lyotropic phase at high concentrations. In the case of polymer **4**, any LC formation was not observed in both CH_3_CN and CH_3_OH but in DMSO. This result suggested that its poor solubility in acetonitrile and methanol results in less interaction between this polymer and solvents. However, due to its high solubility in DMSO, a fully-grown LC texture was observed at a high concentration (50 wt%). Polymer **5** was well-soluble in DMSO and CH_3_OH, but not in CH_3_CN. It showed isotropic solutions as high as 30 wt%, but no biphasic solution in DMSO. Its lyotropic phase occurred at 35 wt% in this solvent. In contrast, it did not form a isotropic solution in CH_3_OH, but exhibited a biphasic solution over a broad range of concentrations (1–30 wt%) in this solvent. This phenomenon may arise from the increased solubility of polymer **5** due to the long aliphatic chain in the counterion. Therefore, the lyotropic properties of polymer **5** were found to be at relatively high concentrations (35 wt% in DMSO and 40 wt% in CH_3_OH). As representative examples, Figure 3 displays photomicrographs of polymer **1** at 30 wt% in DMSO, polymer **2** at 20 wt% in CH_3_CN, polymer **3** at 30 wt% in CH_3_CN, and polymer **5** at 40 wt% in CH_3_OH when viewed under cross polarizers exhibiting lyotropic LC Phases. Additional photomicrographs of lyotropic LC phases of these polymers in different solvents are provided in Supplementary Materials (Figure S4). These results are reminiscent of those exhibited by sulfonated poly(phenylene terephalamide)s that exhibit biphasic solutions as low as 1 wt% in water [71,72,73,74].

### 2.4. Lyotropic Properties of Polymer 1 by Small-Angle X-ray Scattering (SAXS)

**Figure 4:**
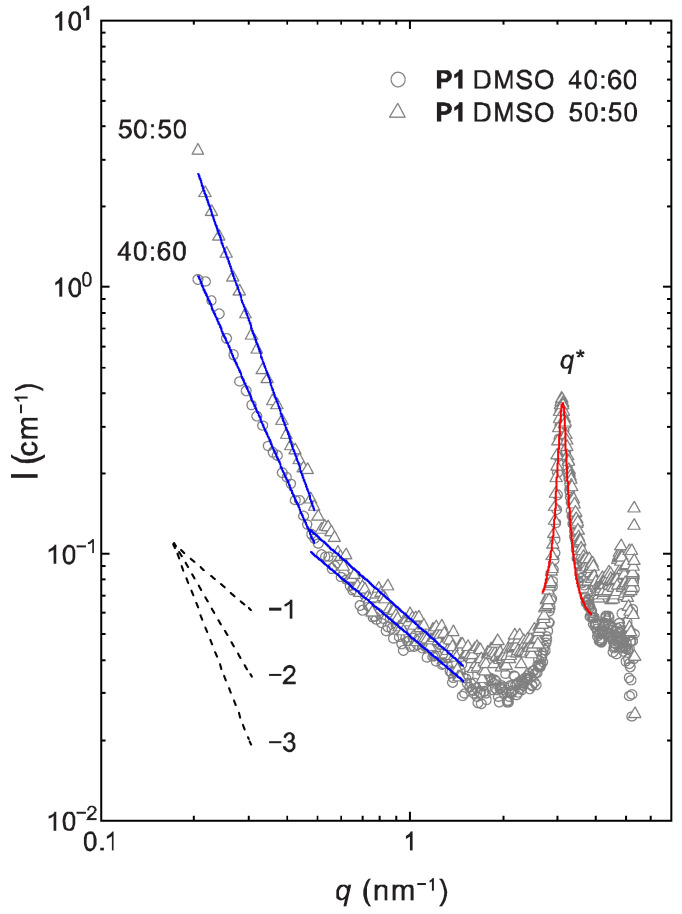
X-ray scattering patterns of polymer **1** in DMSO with polymer: solvent ratios 50:50 (triangles) and 40:60 (circles). Blue and red lines are fit to the power-law regions and to the interference maximum. Black dashed lines show *q*^−1^, *q*^−2^, and *q*^−3^ slopes for comparison.

**Figure 5:**
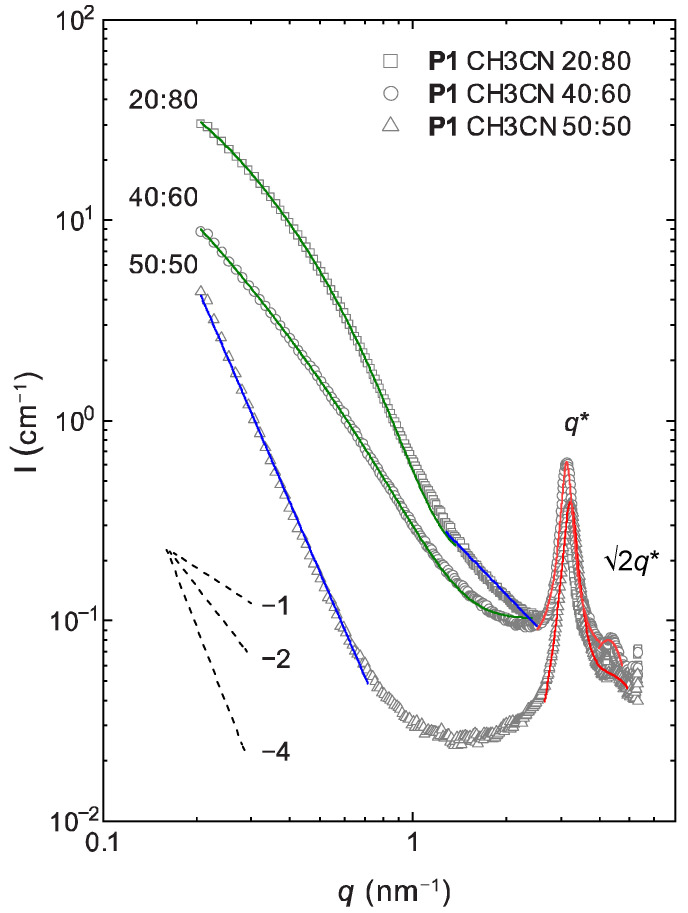
X-ray scattering patterns of polymer **1** in CH_3_CN with polymer: solvent ratios 50:50 (triangles), 40:60 (circles), and 20:80 (squares). Blue, green, and red lines are fit to the power-law sections and to the interference maxima. Black dashed lines show *q*^−1^, *q*^−2^, and *q*^−4^ slopes for comparison.

**Figure 6:**
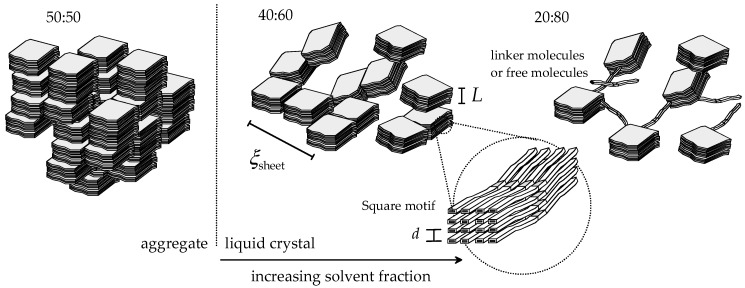
Schematics of polymer **1** in CH_3_CN with increasing polymer-solvent ratio. High polymer fraction (left) promotes aggregation, whereas increasing solvent content facilitates the transition to the lyotropic smectic (and potentially nematic) phase where the size of 2-dimensional objects decreases and linker (or free) molecules appear with further increase. All phases contain smaller square packing motifs regardless of the solvent fraction.

To study polymer liquid crystallinity in further detail, we took one example and conducted small-angle X-ray scattering (SAXS) experiments around optically observed LC phase regimes (20–50%). We selected polymer **1** as the aspect ratio of rigid segments to its diameter is presumably highest among the studied polymer complexes and probed it over characteristic SAXS dimensions $\left(q=0.2–5.3\;{\mathrm{nm}}^{-1}\right)$. Figure 4 plots scattering curves of polymer **1** in DMSO for two solvent fractions. Figure 5 plots scattering curves for polymer **1** in CH_3_CN with increasing solvent fraction. Figure 6 illustrates our interpretation of the latter data. Table 3 compiles selected structural parameters deduced from the data. The highest 50% polymer fraction was manifested by yellow, paste-like yet deformable material; when the solvent fraction is increased from 50% to 60–80%, the visual appearance changed to honey-like viscous fluid.

All scattering curves are dominated by upturns at lower scattering angles, which are followed by interference maxima (Figure 4 and Figure 5). The upturns may be divided into two decaying power-law regions $\left(I\left(q\right)\propto q^{-\alpha }\right)$. In the case of the samples solvated in DMSO, the lower one $\left(0.2–0.5\;{\mathrm{nm}}^{-1}\right)$ decays with a power-law exponent between −2 and −3, whereas the higher one $\left(0.5–1.5\;{\mathrm{nm}}^{-1}\right)$ decays according to −1. We associate the latter decay with several nanometers long polymer sections which appear rigid and locally isolated. Their length is much below the approximately 180 nm total polymer length, estimated from the molecular weight and repeating units. This implies that the rigid sections overlap and become an entangled maze or an aggregate, and this gives rise to the former decay at lower *q*. The conspicuous interference maximum refers to weak crystalline order within aggregates.

Samples containing CH_3_CN also showed the above-described power-law behavior; however, with marked differences (Figure 5). The scattering intensity increased with the increasing solvent fraction. When the solvent fraction was low (50:50), the low *q* range was dominated by the *q*^−4^ decay. This observation points to the Porod scattering arising from particles with sizes exceeding our observation window (>>30 nm).

When the solvent fraction is increased, the data decays as *q*^−2^. This is attributed to a smectic phase that agrees with the micrographs and the visual change observed by the naked eye. The characteristic size of observed sheets or platelets, obtained from the least-squares fitting of the respective scattering range (see Methods section), decreases from about 10 nm to 6 nm, they become marginally thicker, and their aspect ratio decreases from 3 to 1 when the solvent fraction is increased from 60% to 80%. This may point to a further transition from the smectic to the nematic phase. At the same time, the mid-*q* data shows a second power-law section with exponent *α* = 1.6 ≈ 5/3. This exponent corresponds to classic Debye chains, and this observation hints at polymer chains or polymer bundles that are either free or connect LC domains as “tie molecules” or “linker molecules”. The idea of such linker molecules is supported by the high polymer concentration combined with the polymer length exceeding the length of observed polymer sections (and the correlation length).

All curves show distinctive interference maxima at 3–3.2 nm^−1^ and 4.25–4.5 nm^−1^. As these peaks are related as q* and $\sqrt{2}\cdot q*$ this indicates a square (Sq) polymer packing with 2 nm periodicity. We note that this packing motif is not as common as lamellae or hexagonal or monoclinic polymer packing but has been previously found for supramolecular polypyridium salt [12]. The observed maximum shifted towards lower *q* (thus a larger periodic distance) with an increasing solvent fraction, which may be attributed to the solvent molecules incorporating into the ordered motif.

### 2.5. Thermal Properties of Polymers ***1***–***5***

**Figure 7:**
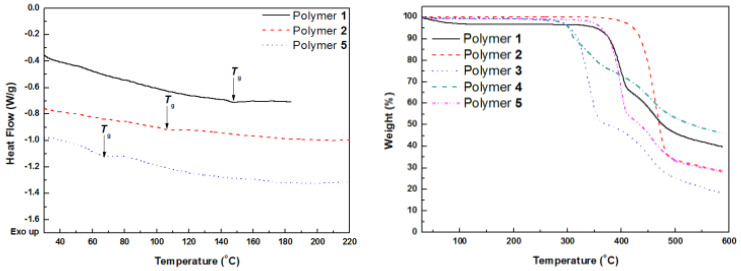
(**Left**) differential scanning calorimetry (DSC) thermograms of the polymers **1**, **2**, and **5** and (**Right**) TGA thermograms of the polymers **1**–**5** obtained at a heating rate of 10 °C/min in nitrogen.

For the fabrication and processing of polymers, amorphous materials are a particularly interesting class of materials for various optoelectronic applications [15]. Therefore, thermal properties of the synthesized ionic polymers **1**–**5** were evaluated by differential scanning calorimetry (DSC) and TGA measurements (Figure 7). Polymer **1** displayed a glass transition temperature (*T*_g_) at 148 °C and one endotherm at 190 °C (not shown), which was attributed to a loss of entrapped solvent molecules as supported by the TGA analysis (Figure 7). Change of the counterion from tosylate to triflimide, polymer **2** exhibited a significantly low *T*_g_ at 107 °C, which was lower by 41 °C when compared with polymer **1**. This decrease in *T*_g_ was presumably related to the fact that the bulky size of triflimide counterion may act as a plasticizer between the polymer chains. This phenomenon is in excellent agreement with the analogous poly(pyridinium salt)s [4,5,6,7,9,10,11,17,19] as well as ionic liquids [75]. Unlike polymer **2**, *T*_g_s of polymers **3** and **4** could not provide any meaningful information regarding their *T*_g_s. However, note here that *T*_g_s of polymers steadily decreased with the size of counterions along with the series, from 148 °C for polymer **1** to 67 °C for polymer **5**. These results indicated that the sizes of counterions resulted in a significant increase in the segmental mobility of the polymer chains. The TGA plots in Figure 7 show that the thermal stability was dependent on the chemical structures of organic counterions. However, polymer **2** has the highest thermal stability among the polymers studied, as expected.

### 2.6. Optical Properties of Polymers ***1***–***5***

#### 2.6.1. UV-Vis Absorption and Emission Spectra of Polymers **1**–**5** in Organic Solvents

One of the interesting characteristics of π-conjugated polymers and π-conjugated polyelectrolytes is their optical properties in solution and solid states. The poly(pyridinium salt)s-fluorene **1**–**5** synthesized in this study exhibited good solubility in common organic solvents, including chloroform and tetrahydrofuran (THF). Therefore, the optical properties of the polymers were evaluated by UV-Vis and photoluminescent (PL) spectrometers. The major absorption spectra of fluorene homopolymer are at λ_max_ 373 nm in chloroform and 381 nm in THF, respectively [40,41,42,43,44]. These absorption peaks were not observed in the UV-Vis results of polymers **2**, **3**, or **5** in these solvents (not shown). These absorption properties indicated that the incorporation of poly(pyridinium salt)s into the polyfluorene chain introduced a disorder of polymer packing in the solution state resulting in a blue shift. Moreover, polymers **1**–**5** showed essentially identical λ_max_ in the range of 340–351 nm various organic solvents such as DMSO, acetonitrile, methanol, THF, or chloroform as detected in their absorption spectra. In general, all the polymers had relatively high molar absorption coefficients in these solvents as measured from the respective Beer-Lambert plots. For example, these values were 83,630 and 82,086 M^−1^cm^−1^ for polymers **1** and **5** in methanol, and that was 79,331 M^−1^cm^−1^ for polymer **2** in CH_3_CN, as shown in Table 4, which are in excellent agreement with the results of a conjugated poly(pyridinium salt) [76,77]. These absorption spectra could be an indication of closely spaced π-π^*^ transitions of conjugated polymer structures. In other words, the interactions between various organic solvents and the chemical structures of polymers did not cause any significant changes in the energies of their ground states. The optical band gaps (E_g_) of the polymers as determined from the onset of wavelength (low energy region) in the UV-Vis absorption spectra are summarized in Table 4.

The light emission spectra of the polymers **1**–**5** were recorded in various organic solvents and are shown in Supplementary Materials (Figure S5). In DMSO, there was no significant difference in their emission spectra that suggested fluorescence was not affected by the counterions. In contrast, a hypsochromic effect was observed by changing the polarity of the solvents from DMSO to THF. Polymer **2** exhibited λ_em_ = 534 nm at an excitation wavelength of 383 nm in DMSO; whereas λ_em_ = 517 nm at an excitation wavelength of 341 nm in THF. Like polymer **2**, polymer **3** exhibited λ_em_ = 533 nm at an excitation wavelength of 347 nm and λ_em_ = 493 nm at an excitation wavelength of 344 nm in DMSO and THF, respectively. However, in case of the polymer **4**, only emission spectra at λ_em_ = 538 and 527 nm at excitation wavelengths at 357 and 344 nm in DMSO and MeOH, respectively, were recorded, but in other solvents, its emission spectra could not be measured due to its poor solubility in these solvents. These data implied a positive solvatochromism phenomenon, that is, the λ_em_ peaks were shifted to the longer wavelengths with the increase in solvent polarity. Like polymers **1**–**3**, polymer **5** followed a similar trend in their PL properties. From the results of the optical studies, the fluorescence in the solution state was dependent on the polarity of the solvents, not on the chemical structures of the counterions. The measured absolute quantum yields (AQYs) of polymers **1**, **2**, and **5** in methanol or acetonitrile were rather low (Table 4), which are still much higher than the relative quantum yield (0.0088) of conjugated poly(pyridinium salt) based of benzidine moiety measured in N,N-dimethylformamide [76,77]. On the other hand, these AQYs were lower than the relative quantum yields (0.10 and 0.18) of non-conjugated poly(pyridinium salt)s with stilbene moiety as a pendent group and dodecamethylene and 4,4′-dicyohexylmethylene units measured in THF [78]. We reported the poly(pyridinium salt)s based on both quinoline and bisquinoline diamine moieties showed relative quantum yields 0.14 and 0.15, respectively, measured in methanol [17,20]. Additionally, sulfonated poly(phenylene vinylene)—a π-conjugated polyelectrolyte—has a quantum yield of 0.01 measured in water [79,80]. Despite that the quantum yields of polymers are solvent-dependent, these results were qualitatively indicative of the fact that the polymers **1**–**5** had good light-emitting properties and emitted green lights (517–535 nm) both in polar aprotic and protic solvents as well as nonpolar solvents. Even polymers **3** and **5** also emitted blue light (493 and 497 nm) in THF. It has been reported in the literature [36] that the poly(pyridinium salt) based on phenanthridine diamine moiety emitted blue light (403–438 nm) in THF and DMSO but emitted green light (516 nm) in water. Interestingly, the water solubility of this polymer has been demonstrated to be used as a new fluorescent probe for the label-free detection of DNA. These results also suggest that the light emission properties of this class of poly(pyridinium salt)s (conjugated and non-conjugated), that is, λ_em_s, quantum yields, and solubility in organic solvents and water depend on the chemical structures of pyridinium salt, counterion (organic and inorganic), aliphatic or aromatic diamine or heterocyclic aromatic diamine moieties.

#### 2.6.2. Emission Properties of Polymers **1**–**5** in the Solid State (Film and Powdered)

**Figure 8:**
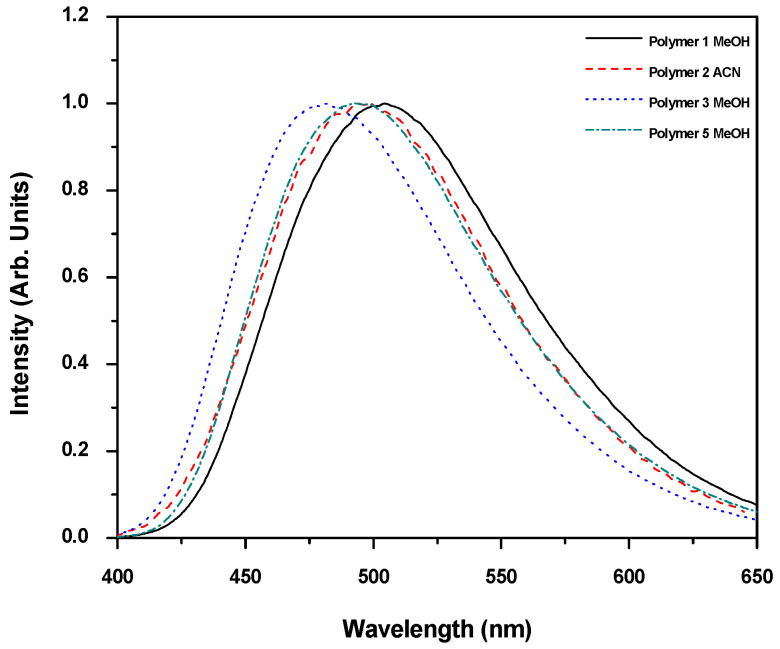
Emission spectra of polymers **1**, **2**, **3**, and **5** in thin films cast from methanol or acetonitrile at various excitation wavelengths.

To evaluate the optoelectronic applications of the polymers, the light-emitting properties of the polymers except polymer **4** in their drop-cast film states were studied. Their λ_em_ peaks were located at 503, 495, 480, and 493 nm at various excitation wavelengths (250–392, 247–336 nm), as displayed in Figure 8. Their emission peaks were independent of their excitation wavelengths. Compared with their solutions spectra, their λ_em_ peaks were found to exhibit hypsochromic (blue) shifts of 25, 38, 47, and 35 nm, respectively. The emission properties may be attributed to the fact that less ordered structures were built up during the film formation resulting in less π-π stacking in the solid-state of these polymers, which are in excellent agreements with the previously reported of this class of poly(pyridinium salt)s [4,5,6,7,9,10,11,16,17,18,19,20,21,22]. The blue shifts in these polymers, as opposed to red shifts in π-conjugated polymers including π-conjugated polyelectrolytes of ordered structures, may be related to the fact that the extensive face-to-face π-π stacking phenomena of aromatic moieties do not occur in these poly(pyridinium salt)s that lead to less ordered structures presumably because of repulsive interactions of positive charges in the backbones of the polymer chains. Note here that both intra- and intermolecular interactions of aromatic moieties are mainly responsible for these ordered structures, which in turn cause to shift λ_em_ peaks bathochromically and to lower the quantum yields of π-conjugated polymers in the solid-state in general [81,82,83,84]. These data suggest that they emitted blue light in the film states cast from organic solvents, and these λ_em_ peaks were affected by the chemical structures of the counterions. Furthermore, the full-width at half-maximum (fwhm) values of PL spectra in the solution and film states were very broad, suggesting that their light-emission stemmed from several chromophoric species. In contrast, synthesized polymers **1**–**5**, except polymer **4,** emitted green light when excited at 354, 360, 355, and 352 nm, respectively (Figure S6). The photomicrographs of thin films **1**, **2**, **3**, and **5** under natural light and UV light are provided in Figure S7, and those of synthesized polymers 1–5 under natural light and UV light are provided in Figure S8, respectively.

#### 2.6.3. Emission Properties Polymers **1**, **2**, and **5** in Organic Solvents-Water Mixture to Study Aggregation Behaviour

**Figure 9:**
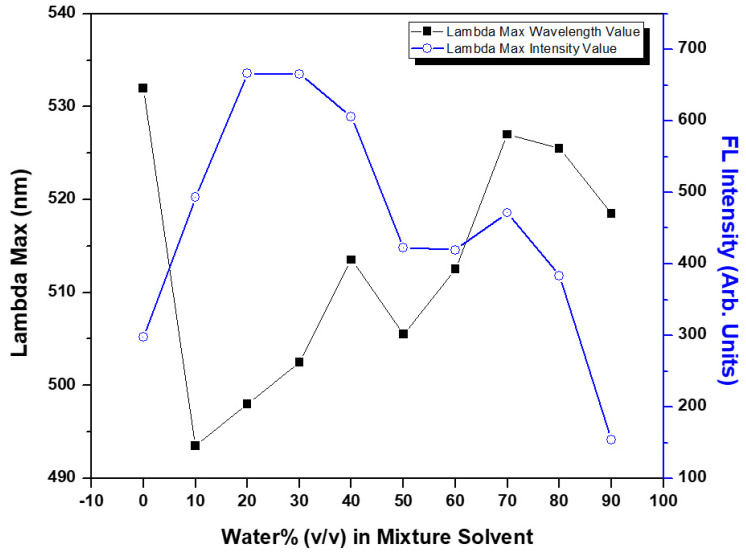
Fluorescence intensity and emission peak of polymer **1** as a function of water content in DMSO (1 μM repeating units, λ_ex_ at 339 nm).

**Figure 10:**
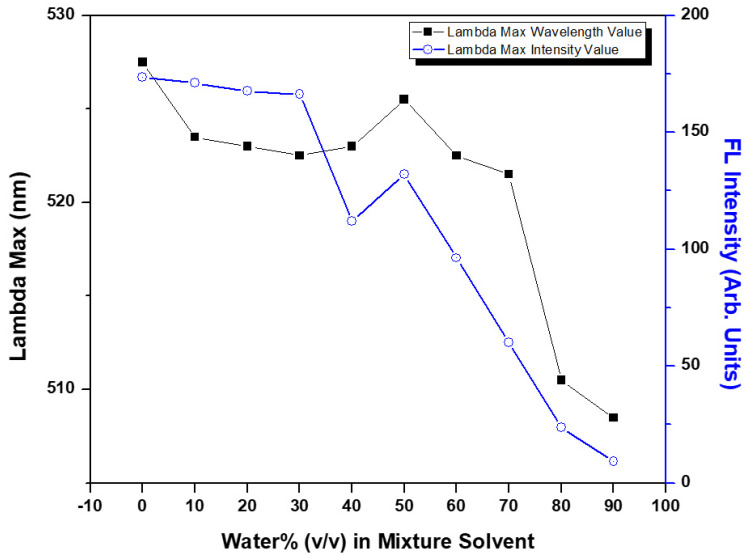
Fluorescence intensity and emission peak of polymer **1** as a function of water content in CH_3_OH (1 μM repeating units, λ_ex_ at 343 nm).

We also studied fluorescence properties of polymer **1** in a mixture of DMSO/H_2_O and CH_3_OH/H_2_O, **2** in a mixture CH_3_CN/H_2_O, and **5** in a mixture CH_3_OH/H_2_O, since the photophysical properties of poly(pyridinium salt)s in aqueous medium is essential to decipher the interactions of biological molecules such as proteins and DNAs. We examined the fluorescence of polymer **1** in DMSO with varying amounts of water (*v*/*v*, 0–90%), and their emission spectra are displayed in Figure S9. As shown in Figure 9, the emission peak blue shifted by 14 nm, from 532 nm to 518 nm, when water content increased from 0% to 90% (*v*/*v*). In contrast, the emission intensity changed in a complicated manner. When the water content increased from 0% to 20% (*v*/*v*), emission intensity increased gradually; the 30% water content remained unchanged. With further increase in water content to 50%, the emission intensity decreased gradually, and the 60% water content remained unchanged. The intensity was increased at 70% water content, after which it decreased sharply up to 90% water content. The emission peak is dominated by the effect of intramolecular charge transfer and the conformation of the polymer chain, whereas the emission intensity is mainly contributed to by the synergistic effect of polymer aggregates and the viscosity of the mixture solvent [36,85,86]. Polymers tend to form aggregates in water because of interchain hydrophobic interactions. These aggregates cause the quenching of the fluorescence of the polymer and consequently lowers emission intensity. In a mixture of DMSO/H_2_O, a low water content solution is the more viscous medium, resulting in high fluorescence intensity by inhibiting the nonradiative decay of the polymer in the excited state. The inhibition of nonradiative decay occurs because the rotation and distortion of the polymer chain are hindered in a viscous medium. Interestingly, polymer **1** did not precipitate out from DMSO (good solvent) with the addition of the high amount of poor solvent water (90%). This polymer showed λ_em_ peak at 518 nm in water that was a blue shift when compared with that in DMSO. In contrast, the poly(pyridinium salt) based on phenanthridine diamine moiety showed λ_em_ peak at 516 nm in water that was a red shift when compared with that (438 nm) in DMSO [36]. Figure S10 shows the emission spectra of polymer **1** in methanol with varying amounts of water (*v*/*v*, 0–90%). The emission spectrum of this polymer in methanol was exceedingly broad which in contrast to the emission spectrum of sulfonated poly(phenylene ethynylene), PPE-S, in this solvent [80]. As shown in Figure 10, the emission peak blue shifted by 20 nm, from 528 nm to 508 nm, when water content increased from 0% to 90% (*v*/*v*), which was in stark contrast to the PPE-S in water [80]. With the addition of water up to 30%, emission intensity was decreased slightly, dropped precipitously with 40% water, increased with 50% water, and after which dropped precipitously with the further addition of water up to 90%. The emission intensity was not increased with low water content, as opposed to the DMSO mixture, which was related to the less viscous medium. With the addition of more water, a precipitous decrease in emission intensity occurred because the aggregates formed quenched the fluorescence. In methanol, it showed a larger blue shift than that in DMSO. It did not precipitate out from methanol with the addition of a large amount of water (*v*/*v*, 90%).

Figure S11 shows the emission spectra of polymer **2** in acetonitrile with varying amounts of water (*v*/*v*, 0–90%). Like those in polymer **1** in DMSO and methanol, the emission spectrum of polymer **2** in acetonitrile was exceedingly broad. As shown in Figure S12, the emission peak blue shifted by 32 nm, from 534 nm to 502 nm, when water content increased from 0% to 90% (*v*/*v*). The emission intensity gradually decreased with the addition of water as the aggregates started to grow. Like polymer **1** in methanol/water, this decrease in emission intensity was related to aggregation-induced quenching. It also did not precipitate out from acetonitrile with the addition of a large amount of water (*v*/*v*, 90%).

Figure S13 shows the emission spectra of polymer **5** in methanol with varying amounts of water (*v*/*v*, 0–90%). Like those in polymer **1** in DMSO and methanol and polymer **2** in acetonitrile, its emission peak was exceedingly broad in this solvent. As shown in Figure S14, the emission peak blue shifted by 8 nm, from 528 nm to 520 nm, when water content increased from 0% to 90% (*v*/*v*). Emission intensity decreased precipitously with the addition of water up to 50% (*v*/*v*) during which aggregates started to form. With the further addition of 90% water, emission intensity decreased gradually because, as expected, aggregates caused quenching of fluorescence. Polymer **5** also did not precipitate out from methanol with the addition of a large amount of water (*v*/*v*, 90%). The formation of aggregates other π-conjugated polyelectrolytes in water is widespread in the literature. It is likely that the extensive π-π interactions of the backbone and electrostatic interactions of ionic groups in the side chain with the solvents, among other factors, are responsible for the aggregates [55,87,88]. This mechanism is also supported by the theoretical molecular dynamics simulations of the aggregation of 9,9′-bis(4-phenoxy-butylsulfonate) fluorene-2,7-diyl and 1,4-phenylene tetramer in water [89]. In the present study, the extensive π-π interactions of the conjugated polymer backbone and electrostatic interactions of ionic groups in the main chain with the solvent, among other factors, were responsible for the formation of aggregates.

## 3. Materials and Methods

### 3.1. Instrumentation

The FTIR spectra were recorded with a Shimadzu infrared spectrometer (Shimadzu Scientific Instruments, Inc., Moorpark, CA, USA). Polymer samples were prepared by coating NaCl plates with various polymers and subsequently vacuum dried at 70 °C overnight. The ^1^H and ^19^F and spectra were obtained using VNMR 400 spectrometer (Varian Inc., Palo Alto, CA, USA) operating at 400 MHz for ^1^H and 376 MHz for ^19^F with three RF channels at room temperature, and chemical shifts were referenced to tetramethylsilane (TMS) for proton nuclei and trichlorofluoromethane (CFCl_3_) for fluorine nuclei. The NMR samples were prepared by applying gentle heating to dissolve the polymer in *d_6_*-DMSO. Elemental analysis was performed by Atlantic Microlab Inc. (Norcross, GA, USA). The GPC analyses of polymers were conducted using a VISCOTEK chromatograph (Malvern Panalytical Inc., Westborough, MA, USA) equipped with three ViscoGel1-MBHMW-3078 columns and a tetra detector array (UV/visible, low- and right-angle light scattering, refractive index, and viscometer) at 50 °C. DMSO containing 0.01 M LiBr was the mobile phase, and the flow rate was set at 0.7 mL/min. The instrument was calibrated using a pullulan standard of P-50. Lyotropic liquid crystal (LC) properties of the polymers were obtained using a polarized optical microscope (POM, Nikon, Model Labophot 2) equipped with crossed polarizers. Samples of polymers for lyotropic LC properties were made by dissolving known amounts of polymer into known amounts of organic solvents (DMSO, CH_3_CN, or MeOH). Differential scanning calorimetry (DSC) measurements of polymers were conducted on TA module DSC Q200 series (TA Instruments, New Castle, DE, USA) in nitrogen at heating and cooling rates of 10 °C/min. The temperature axis of the DSC thermograms was calibrated before using the reference standard of high purity indium and tin. Thermogravimetric analyses (TGA) of polymers were performed using a TGA Q50 instrument (TA Instruments, New Castle, DE, USA) in nitrogen. The TGA data were collected at temperatures between 30 and 600 °C at a heating rate of 10 °C/min. The UV-Vis absorption spectra of polymer solutions in organic solvents were recorded at room temperature using Varian Cary 50 Bio UV-Visible spectrophotometer (Agilent Technologies, Santa Clara, CA, USA) in quartz cuvettes. Photoluminescence spectra in solutions and thin films were recorded with a Perkin–Elmer LS 55 luminescence spectrometer (Perkin Elmer, Akron, OH, USA) with a xenon lamp light source. Absolute quantum yields of polymers **1**–**5** in both methanol solution and powdered form were measured with a Horiba Fluorolog fluorimeter (HORIBA Instruments Inc., Irvine, CA, USA) equipped with an integrating sphere.

### 3.2. Materials

Lithium triflimide, methyl orange, dodecylbenzene sulfonic acid (sodium salt), dioctyl sulfosuccinate (sodium salt), and common organic solvents were purchased from Sigma-Aldrich (Milwaukee, WI, USA) and TCI America (Portland, OR, USA) and were used without further purification. For synthesis and purification purposes, reagent grade solvents including acetonitrile, ethanol, methanol, ethyl acetate, spectral grade dimethyl sulfoxide (DMSO), acetonitrile, methanol, chloroform, and tetrahydrofuran were used as obtained from Sigma-Aldrich. Deuterated solvents were obtained from Cambridge Isotope Laboratories, Inc. (Tewksbury, MA, USA). The 4,4′-(1,4-phenylene)bis(2,6-diphenylpyrylium *p*-toluene sulfonates), **M**, [5] and 9,9′-dioctyl-9*H*-fluorene-2,7-diamine were synthesized (DSC melting peak maximum at 69 °C, mp = 58–63 °C) [23] according to the reported procedures [5,23].

### 3.3. Synthesis of Polymer ***1***

The bis(pyrylium salt)s, **M**, (5.00 g, 5.65 mmol), 9,9′-dioctyl-9*H*-fluorene-2,7-diamine (2.38 g, 5.65 mmol), and DMSO (120 mL) were placed in a 250 mL three-necked round bottom flask equipped with a magnetic stirrer. A small amount of toluene (6 mL) was added to remove the water generated during the reaction by azeotrope, which was facilitated by using a Dean-Stark trap and water condenser. The solution was stirred at 130–135 °C for 48 h under a nitrogen atmosphere. After the reaction was completed, the solution was concentrated by a rotary vapor until the solution became viscous. It was then poured into water to precipitate the polymer. The precipitated solid was washed with water several more times to remove any residual impurities and collected by vacuum filtration. The collected polymer was additionally washed a few times with boiling water and dried *in vacuo* at 110 °C for 48 h [5]. Data for polymer **1**: Anal. Cacld for C_83_H_82_N_2_O_6_S_2_ (1267.68): C, 78.64; H, 6.52; N, 2.21; S, 5.06. Found: C, 76.67; H, 6.62; N, 2.27; S, 4.79; FTIR (film, cm^−1^) ν = 3047 (=C–H aromatic stretching), 1620–1448 (C=C and C=N aromatic ring stretching), 1196 (C–N^+^), 1119 (S=O asymmetric stretching), and 1033–1010 (S=O symmetric stretching).

### 3.4. Synthesis of Polymers ***2***–***5***

Polymers **2**–**5** were synthesized through a metathesis reaction from the respective polymer **1** with excess appropriate counterion salts in a common organic solvent [5]. In general, polymer **1** (1.00 g, 0.789 mmol) was dissolved in MeOH (50 mL), and a solution of counterion salt (1.73 mmol, 2.2 equivalent based on the polymer ratio) was added into the solution while keeping the temperature at 40 °C. The solution was stirred under reflux for 48 h. After the reaction was completed, the solution was concentrated by a rotary evaporator until it became viscous. The concentrated solution was poured into water to precipitate out the polymer. The solid was washed with water for 2 h to remove any residual impurities, and the reaction step was repeated one or two more times until all tosylate counterions were completely exchanged to the target counterions, which was confirmed from the analysis of the ^1^H NMR spectrum. The purified polymers were dried *in vacuo* at 110 °C for 24 h.

Data for polymer **2**: Anal. Cacld for C_73_H_68_F_12_N_4_O_8_S_4_ (1485.58): C, 59.02; H, 4.61; N, 3.77; S, 8.63. Found: C, 59.29; H, 4.59; N, 3.86; S, 8.43; FTIR (film, cm^−1^) ν = 3063 (=C–H aromatic stretching), 1620–1466 (C=C and C=N aromatic ring stretching), 1350 (C–F stretching), 1196 (C–N^+^), 1119 (S=O asymmetric stretching), and 1057 (S=O symmetric stretching).

Data for polymer **3**: Anal. Cacld for C_109_H_142_N_2_O_14_S_2_ (1768.48): C, 74.03; H, 8.09; N, 1.58; S, 3.63. Found: C, 73.25; H, 7.90; N, 1.65; S, 3.48; FTIR (film, cm^−1^) ν = 3055 (=C–H aromatic stretching), 1736 (C=O stretching), 1620–1458 (C=C and C=N aromatic ring stretching), 1242–1158 (C–N^+^ and S=O asymmetric stretching), and 1034 (S=O symmetric stretching).

Data for polymer **4**: Anal. Cacld for C_97_H_96_N_8_O_6_S_2_ (1534.00): C, 75.95; H, 6.31; N, 7.30; S, 4.18. Found: C, 74.01; H, 6.40; N, 6.96; S, 3.83; FTIR (film, cm^−1^) ν = 3048 (=C–H aromatic stretching), 1620–1458 (C=C and C=N aromatic ring stretching), 1458 (N=N), 1365 (C–N stretching), 1196–1111 (C–N^+^ and S=O asymmetric stretching), and 1026 (S=O symmetric stretching).

Data for polymer **5**: Anal. Cacld for C_105_H_126_N_2_O_6_S_2_ (1576.26): C, 80.01; H, 8.06; N, 1.78; S, 4.07. Found: C, 78.37; H, 8.02; N, 1.87; S, 3.91; FTIR (film, cm^−1^) ν = 3055 (=C–H aromatic stretching), 1620–1458 (C=C and C=N aromatic ring stretching), 1195–1134 (C–N^+^ and S=O asymmetric stretching), and 1034–1011 (S=O symmetric stretching).

### 3.5. X-ray Scattering Experiments

SAXS experiments were conducted at the CREDO facility of the Hungarian Research Centre for Natural Sciences [90,91]. Cu Kα X-rays were produced by a GeniX3D Cu ULD integrated beam delivery system (Xenocs SA, Sassenage, France), and the scattered radiation was detected using a Pilatus-300k CMOS hybrid pixel position sensitive detector (Dectris Ltd., Baden, Switzerland) placed 536 mm downstream from the sample. The accessible *q*-range was 0.2 to 5.3 nm^−1^ calibrated by SBA15 mesoporous silica and silver behenate (here q=(4π/λ)sin(2θ/2) denotes the magnitude of scattering vector, with 2θ denoting the scattering angle and λ the monochromatic X-ray wavelength of Cu Kα radiation, 0.154 nm). The samples were filled into borosilicate glass capillaries of approximately 2 mm outer diameter and 0.01 mm wall thickness and kept at room temperature. Exposures of each sample were repeated in 5 min units with frequent re-measuring of background signals and calibration samples until the desired signal-to-noise ratio was obtained. Each scattering pattern was corrected for sample self-absorption, instrumental background, and detector flatness using the standard procedure implemented in the data collection program. Moreover, the scattering patterns were statistically filtered to remove artifacts from external radiation. The scattering intensity I(q) was transformed to absolute units (differential scattering cross-section) by measuring a glassy carbon sample of known absolute scattering intensity under the same conditions as our samples.

Initial data interpretation was made using a simple scaling argument. In this consideration, I(q) follows a power law $I\left(q\right)\propto q^{-\alpha }$ where the exponent α=4 refers to 3-dimensional particles with a smooth surface (Porod’s law) and α=2 to sheet-like particles. When the exponent falls between 2 and 3, the system may be understood as mass fractal material. When the sample was expected to contain sheet-like particles, the data were fitted to the equation

(1) $$I\left(q\right)\propto \frac{1}{1+\xi_{\mathrm{sheet}}^{2}q^{2}\exp\left(q^{2}L^{2}/12\right)},$$

where $\xi_{\mathrm{sheet}}$ represents the lateral size and L the thickness of sheet. This model and its applicability to LC π-conjugated polymers have been discussed in [92,93].

## 4. Conclusions

Several poly(pyridinium salt)-fluorene polymers **1**–**5** with various organic counterions were prepared by using ring-transmutation and metathesis reactions. Their chemical structures were characterized by FTIR and ^1^H and ^19^F NMR spectroscopic techniques, thermal properties by DSC and TGA, and their LC order by polarized microscopy and SAXS. Their number-average molecular weights (M_n_) were in the range of 96.5–107.8 kg/mol and polydispersity indices in the range of 1.12–1.88 as determined by gel permeation chromatography. *T*_g_s of some polymers were lowered by the introduction of bulky counterions, and the thermal stabilities of the polymers were in the range of 305–423 °C. All polymers exhibited lyotropic liquid-crystalline phases in organic solvents (DMSO, CH_3_CN, or MeOH) above their critical concentrations, depending on the counterions. Polymer **1** was found to show a square packing motif within the observed LC platelets. The analyses of their optical properties revealed that the light emission spectra were dependent on the chemical structures of the counterions in the film states resulting in blue light, and the majority of them emitted green light in organic solvents except in a few cases they emitted blue light in THF. Additionally, they formed aggregated structures in DMSO, CH_3_CN, and CH_3_OH with the addition of water (*v*/*v*, 0–90%) regardless of the chemical structures of the counterions and the emission peaks of these aggregated were blue shifted.

## Figures and Tables

**Table 1:** Molecular weights of poly(pyridinium salt)-fluorenes **1**–**5**.

Polymer	M_n_^a^	M_w_^b^	PDI ^c^	dn/dc (mL/g)	R_h_ (nm) ^d^	R_g_ (nm) ^e^
**1**	96.5	181.8	1.88	0.1417	13.3	50.0
**2**	103.6	155.8	1.50	0.0878	12.7	24.4
**3**	107.8	130.0	1.21	0.0885	12.4	24.2
**4**	96.6	108.1	1.12	0.1089	11.8	33.0
**5**	103.9	124.1	1.19	0.0945	12.2	28.8

^a^ Number-average molecular weight in kg/mol. ^b^ Weight-average molecular weight in kg/mol. ^c^ Polydispersity index (M_w_/M_n_). ^d^ Hydrodynamic radius. ^e^ Radius of gyration.

**Table 2:** Solution properties of poly(pyridinium salt)-fluorenes **1**–**5**.

Polymer	1	2	3	4	5
DMSO	1–10% I ^a^ 20% B ^a^ 30% L ^a^	20% B 30% L	1–30% I 40% L	1–40% I 50% L	1–30% I 35% L
CH_3_CN	10% B 20% L	20% L	1–20% I 30% L	−	−
CH_3_OH	10% B 20% L	−	20% B 30% L	−	30% B 40% L

^a^ I = Isotropic; B = Biphasic (anisotropic + isotropic); and L = Lyotropic.

**Table 3:** Parameters estimated from the fits to the small-angle X-ray scattering (SAXS) data of polymer **1** in DMSO and CH_3_CN: *α* is the power-law exponent, *ξ*_sheet_ and *L* are the size and thickness of the polymer sheets while *d* is the long period deduced from the observed interference maximum.

	Polymer:Solvent Ratio	50:50	60:40	80:20
	Analyzed *q*-range (nm^−1^)			
**P1**:DMSO				
*α*	0.21–0.48	3.36 ± 0.06	2.67 ± 0.05	-
	0.48–1.49	1.03 ± 0.04	0.98 ± 0.03	-
*d* (nm)		2.0 ± 0.1	2.0 ± 0.1	-
**P1**:CH_3_CN				
model		aggregate	sheet	sheet
*α*	0.21–0.71	3.59 ± 0.03	-	-
	1.27–2.50	-	-	1.60 ± 0.03
*ξ*_sheet_ (nm)	0.21–(1.37)–2.42	-	10.15 ± 0.25	5.90 ± 0.08
*L* (nm)		-	3.30 ± 0.07	4.71 ± 0.01
*d* (nm)	2.53–4.00	1.95 ± 0.1	2.0 ± 0.1	2.0 ± 0.1

**Table 4:** Optical properties of poly(pyridinium salt)-fluorenes, **1**–**5**.

Polymer	1	2	3	4	5
UV λ_abs_ (nm) ^a^	347	347	347	351 (429)	347
Band gap (eV)	3.04	3.04	3.04	2.99	3.04
UV λ_abs_ (nm)	343 ^b^	342 ^c^	−	−	343 ^b^
Molar Absorptivity (M^−1^cm^−1^)	ε_343_ = 83,630	ε_342_ = 79,331	−	−	ε_343_ = 82,086
PL λ_em_ DMSO (nm)	534	534	533	538	533
PL λ_em_ CH_3_CN (nm)	535	533	534	−	531
PL λ_em_ CH_3_OH(nm)	528	533	527	527	528
AQY(Φ_F_) ^d^	0.040	0.051	−	−	0.038
PL λ_em_ THF (nm)	−	517	493	−	497
PL λ_em_ CHCl_3_ (nm)	−	524	524	−	518
PL λ_em_ film (nm)	503 ^e^	495 ^f^	480 ^e^	−	493 ^e^
PL λ_em_ (powdered) ^g^	542	529	514	−	517

^a^ Measured in DMSO. ^b^ Measured in methanol. ^c^ Measured in CH_3_CN. ^d^ Absolute quantum yield (methanol or acetonitrile solution) measured by an integrating sphere. ^e^ Thin film cast from CH_3_OH. ^f^ Thin film cast from CH_3_CN. ^g^ As synthesized polymer. PL: photoluminescent; AQY: absolute quantum yields.

## Data Availability

Not applicable.

## Supplementary Materials

The following are available online. Figure S1: FTIR spectra of polymers **1**–**5**, Figure S2: ^19^F NMR spectrum of polymer **2**, Figure S3: ^1^H NMR spectra of polymers **1**–**5**, Figure S4: POM textures of polymers **1**–**4**, Figure S5: Emission spectra of **1**–**5** in different organic solvents, Figure S6: Emission spectra of as synthesized polymers **1**–**3**, **5**, Figure S7: Photomicrographs of polymers **1**, **2**, **3**, and **5** in thin film states under regular light and UV light, Figure S8: Photomicrographs of synthesized polymers **1**, **2**, **3** and **5** (powdered form) under regular light and UV light, Figure S9: Emission spectra of polymer **1** in DMSO/H_2_O, Figure S10: Emission spectra of polymer **1** in CH_3_OH/H_2_O, Figure S11: Emission spectra of polymer **2** in CH_3_CN/H_2_O, Figure S12: Fluorescence intensity and emission peak of polymer **2** as a function of water content in CH_3_CN, Figure S13: Emission spectra of polymer **5** in CH_3_OH/H_2_O, and Figure S14: Fluorescence intensity and emission peak of polymer **5** as a function of water content in CH_3_OH.

## Sample Availability

Samples of the compounds are not available from the authors.
