# Supplementary material for: Poly(Pyridinium Salt)s Containing 2,7-Diamino-9,9′-Dioctylfluorene Moieties with Various Organic Counterions Exhibiting Both Lyotropic Liquid-Crystalline and Light-Emitting Properties
Source: Molecules. 2021 Mar 12;26(6):1560. doi: 10.3390/molecules26061560 (PMC7998704; doi:10.3390/molecules26061560)
Supplement: Supplementary file 1 [file molecules-26-01560-s001.pdf]

Supplementary Materials

Poly(pyridinium salt)s Containing 2,7-Diamino- 9,9′-dioctylfluorene Moieties with Various Organic Counterions Exhibiting both Lyotropic Liquid-Crystalline and Light-Emitting Properties

Pradip K. Bhowmik [1,*], Tae Soo Jo[1], Jung Jae Koh[1], Jongwon Park[1], Bidyut Biswas[1], Ronald Carlo G. Principe[1], Haesook Han[1], András F. Wacha[2], and Matti Knaapila[3]

1 Department of Chemistry and Biochemistry, University of Nevada Las Vegas, 4505 S. Maryland Parkway Box 454003, Las Vegas, NV 89154-4003, USA

2 Institute of Materials and Environmental Chemistry, Research Centre for Natural Sciences, , Magyar tudósok körútja 2, Budapest, 1117 Hungary

3 Department of Physics, Technical University of Denmark, 2800 Kgs. Lyngby, Denmark

* Correspondence: pradip.bhowmik@unlv.edu; Tel.: +1 (702) 895-0885; +1 (702) 895-4072

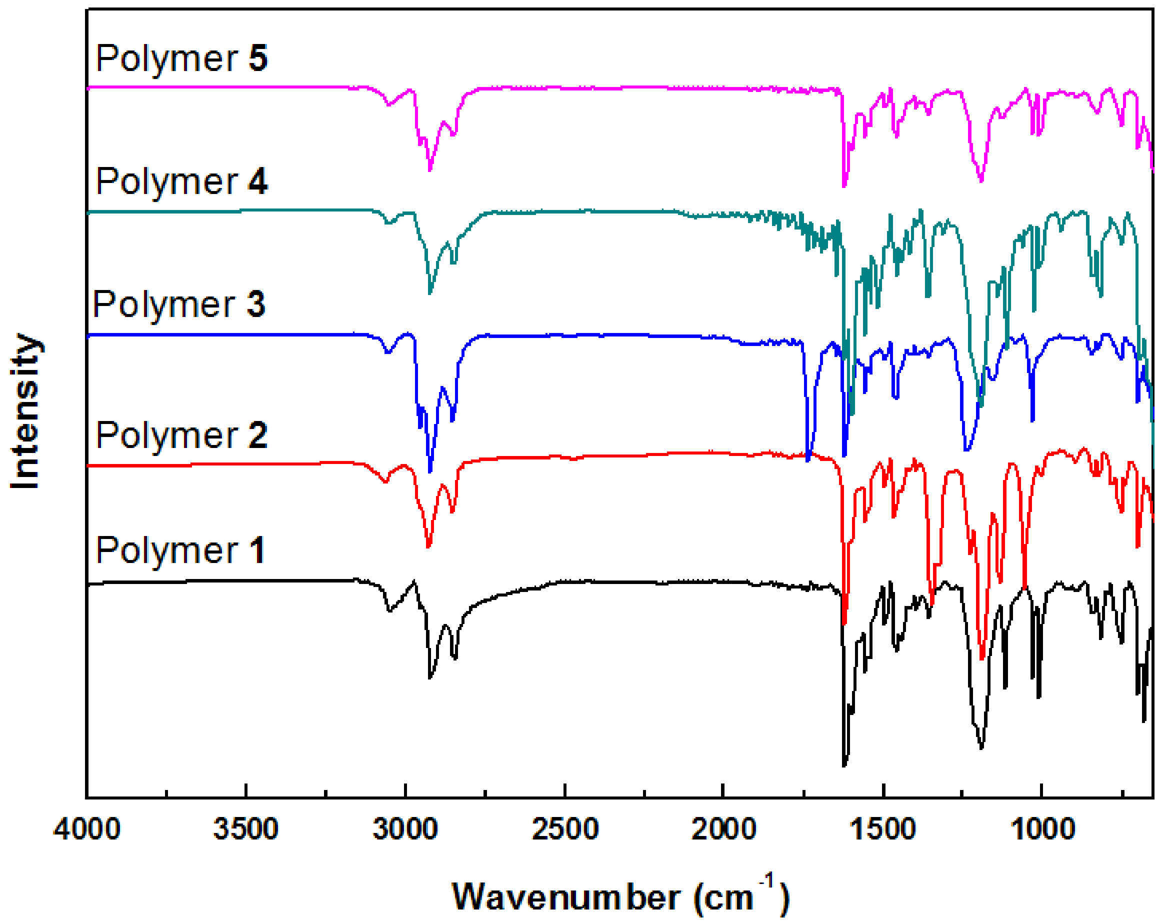

Figure S1. FTIR spectra of polymers **1**–**5** taken at room temperature.

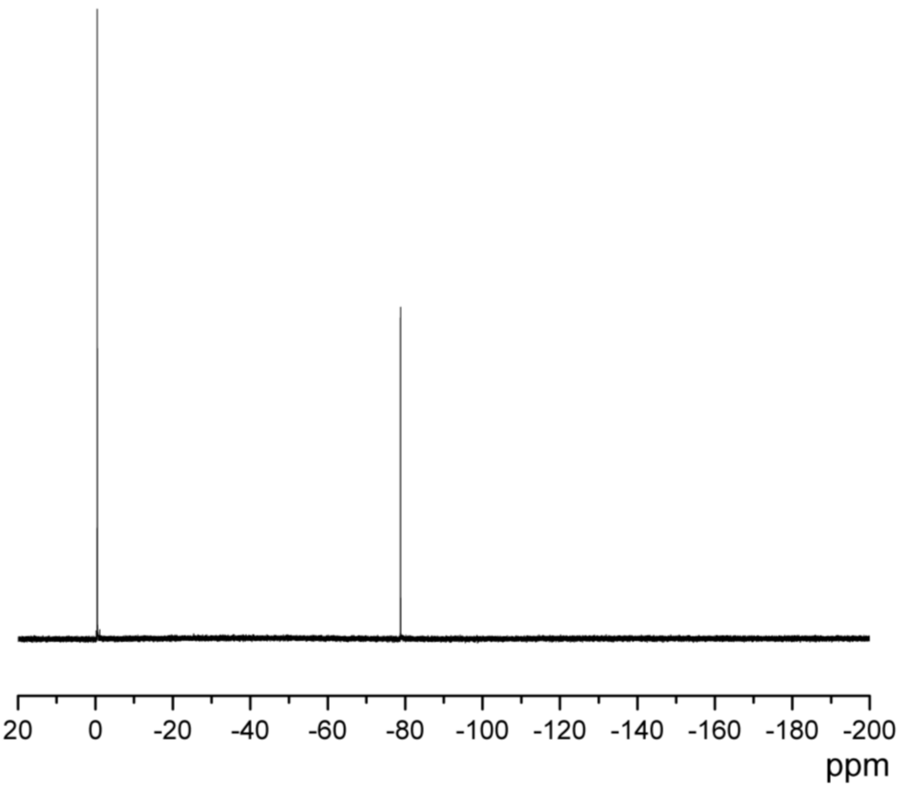

Figure S2. ^{19}F NMR spectrum of polymer **2** in d_6-DMSO at 25 ^oC.

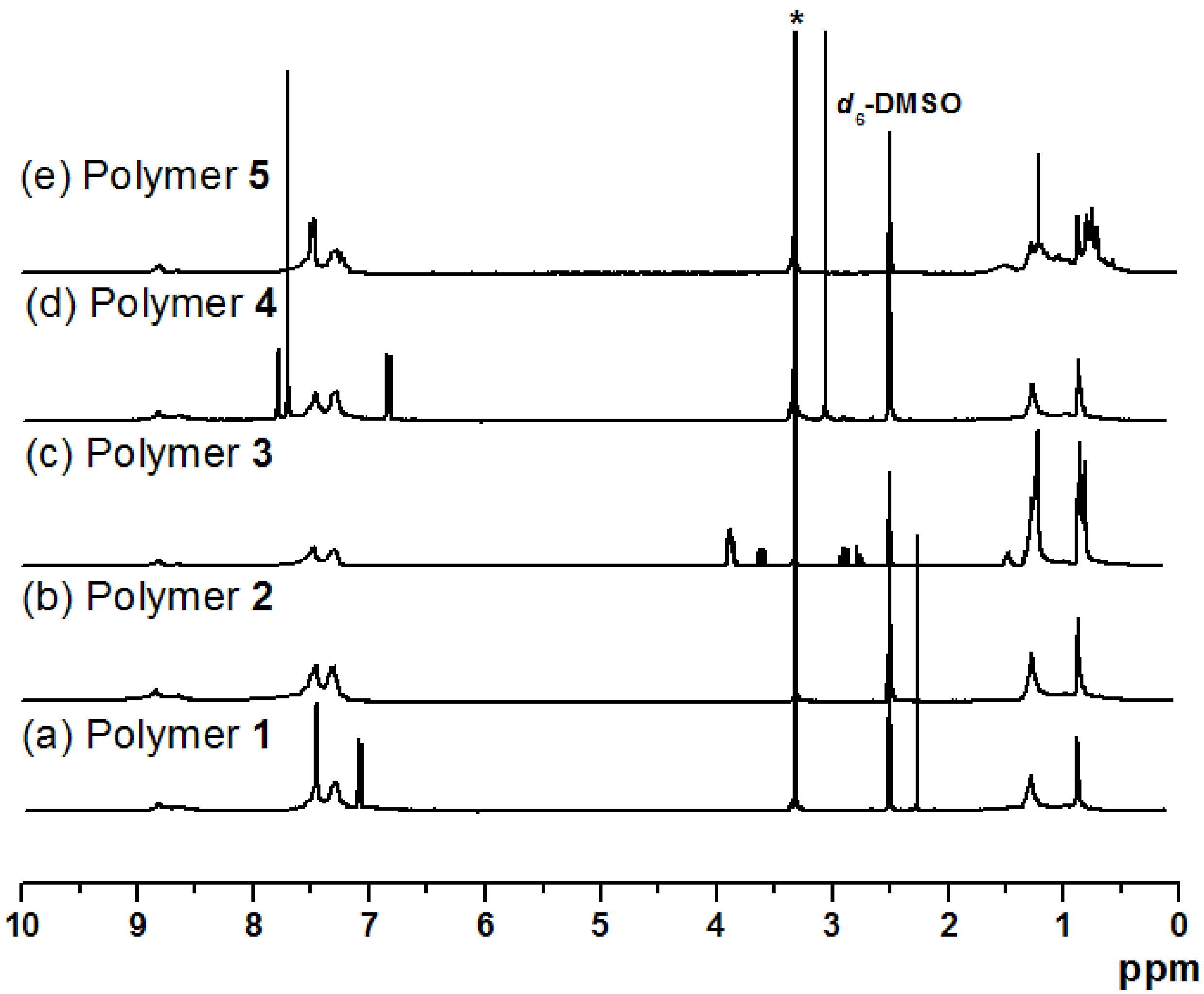

Figure S3. ^{1}H NMR spectra of polymers **1–5** [10 mg/mL in d_6-DMSO at 25 $^{\circ}$C. An asterisk indicates H_2O from the NMR solvent).

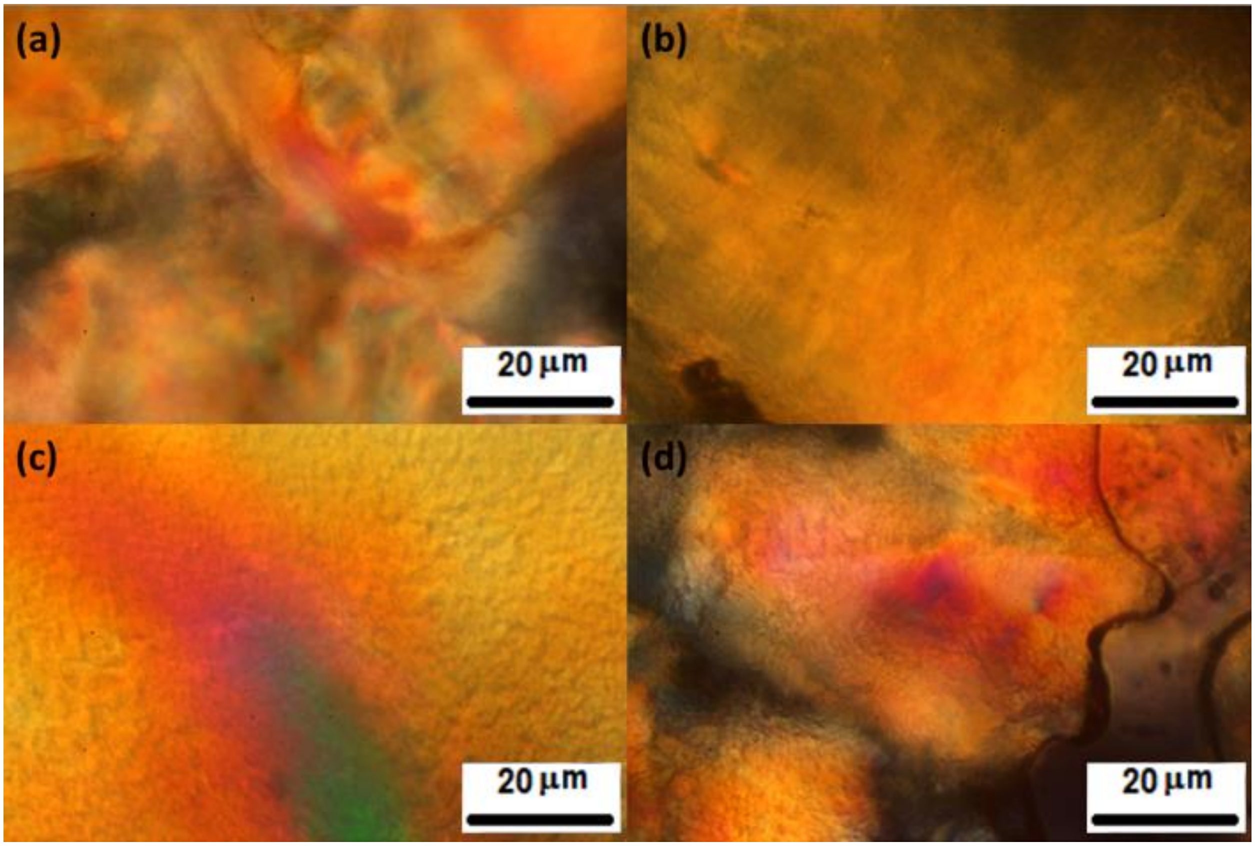

Figure S4. Photomicrographs of (a) polymer **1** at 20 wt % in CH_3OH , (b) polymer **2** at 30 wt % in DMSO, (c) polymer **3** at 40 wt % in DMSO, and (d) polymer **4** at 35 wt % in DMSO under crossed polarizers exhibiting lyotropic LC phase, respectively, (magnification 400x).

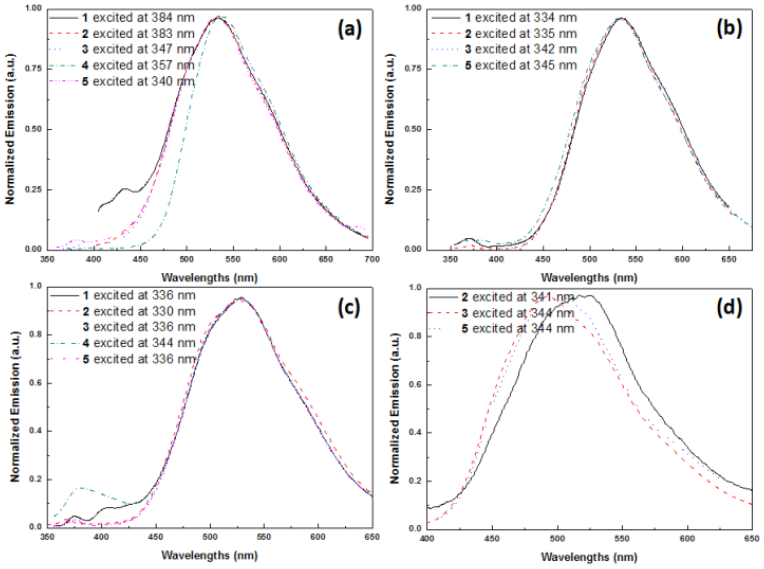

Figure S5. Emission spectra of polymers in (a) DMSO, (b) acetonitrile, (c) methanol, and (d) THF at various excitation wavelengths.

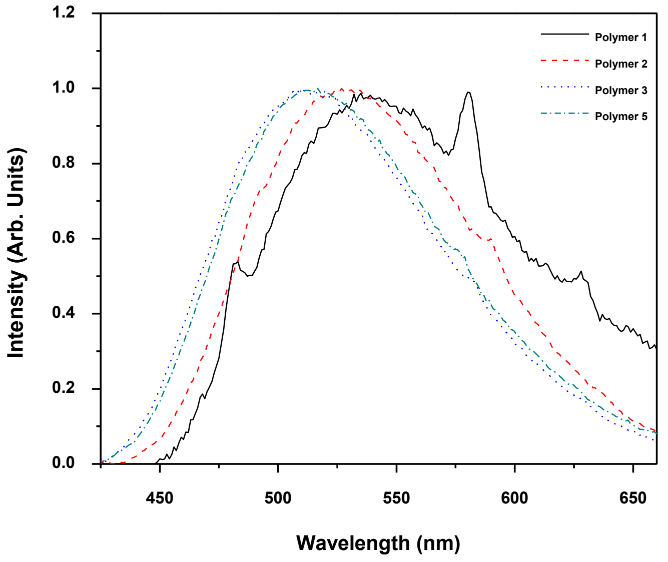

Figure S6. Emission spectra of as synthesized polymers (powdered state) **1-3,** and **5** excited at 354, 360, 355 and 352 nm, respectively.

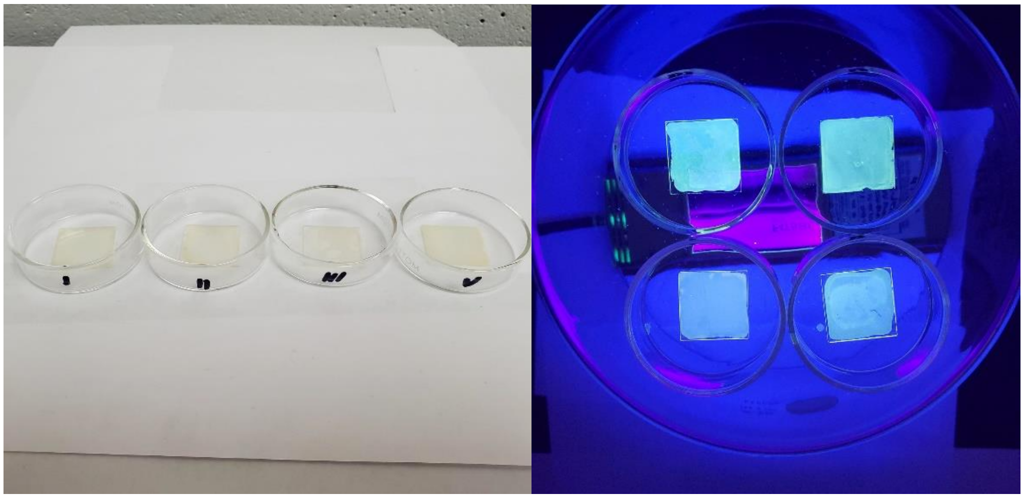

Figure S7. Photomicrographs of polymers **1, 2, 3** and **5** in thin film states under regular light and UV light.

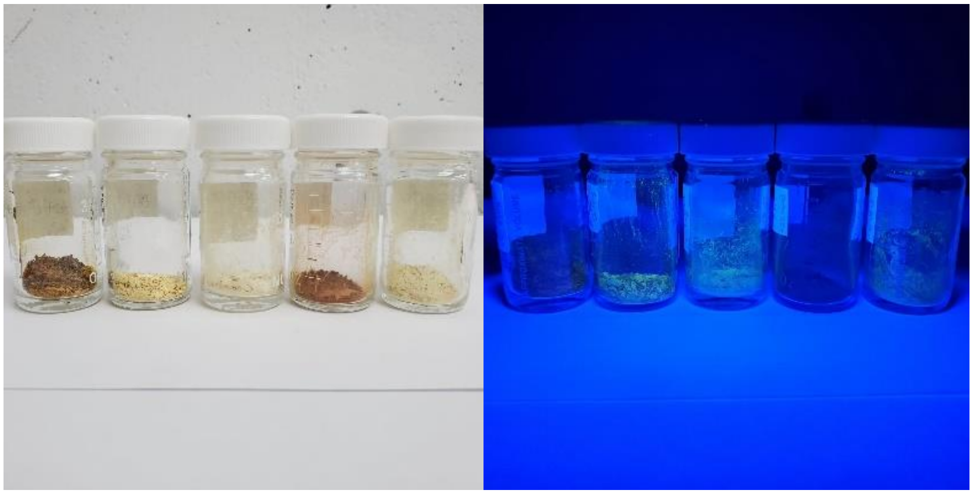

Figure S8. Photomicrographs of synthesized polymers **1-5** (powdered form) under regular light and UV light.

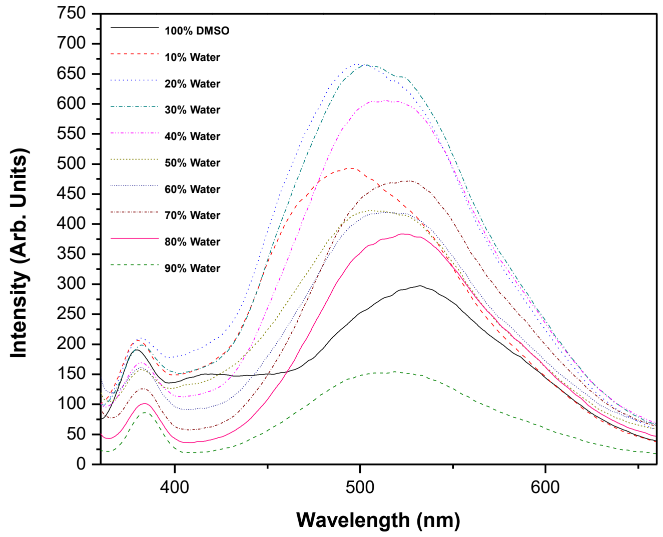

Figure S9. Emission spectra of polymer **1** (1 μM repeating units, excited at 339 nm) in DMSO/H_2O mixtures with varying amounts water % (v/v).

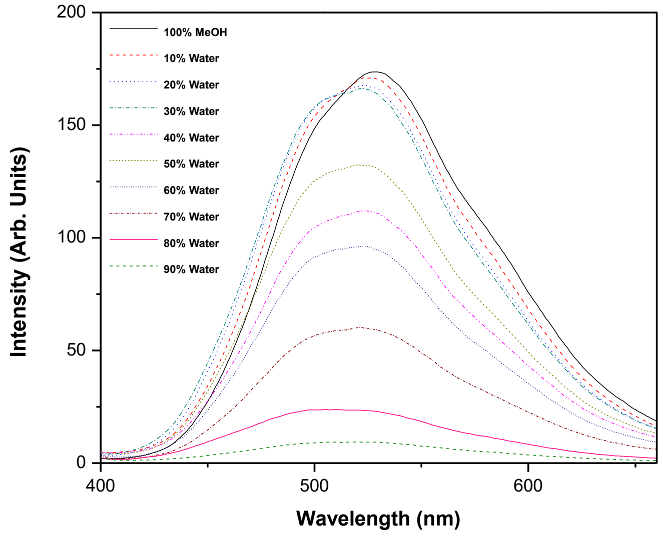

Figure S10. Emission spectra of polymer **1** (1 μM repeating units, excited at 343 nm) in CH_3OH/H_2O mixtures with varying amounts water % (v/v).

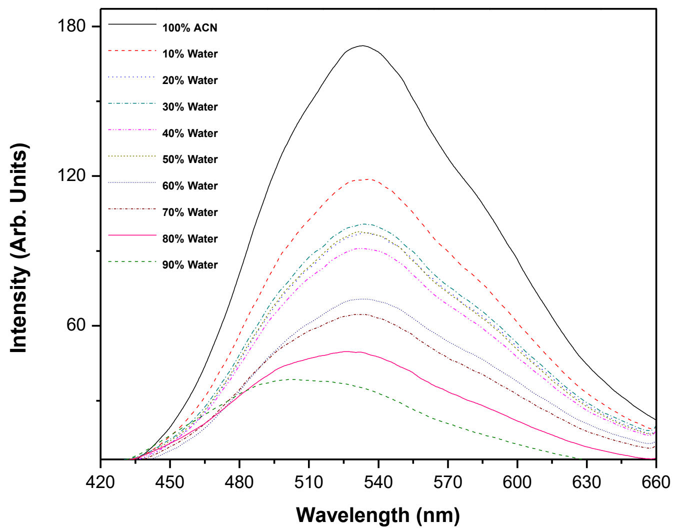

Figure S11. Emission spectra of polymer **2** (1 μM repeating units, excited at 342 nm) in CH_3CN/H_2O mixtures with varying amounts water % (v/v).

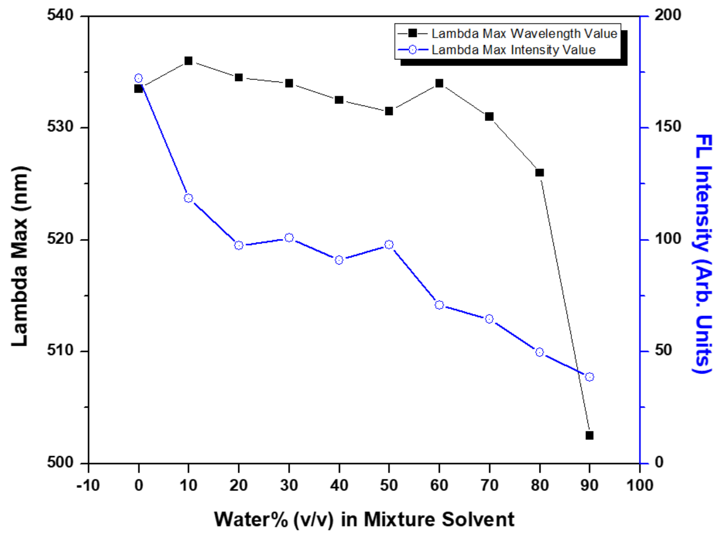

Figure S12. Fluorescence intensity and emission peak of polymer **2** as a function of water content in CH_3CN (1μM repeating units, λ_{ex} at 342).

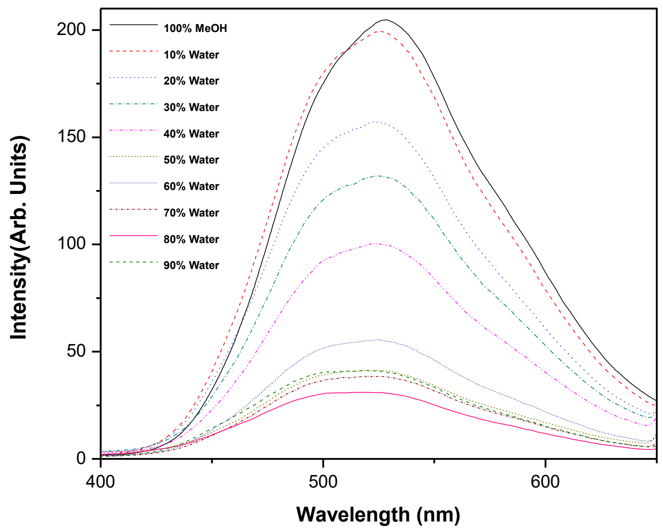

Figure S13. Emission spectra of polymer **5** (1 µM repeating units, excited at 336 nm) in CH_3OH/H_2O mixtures with varying amounts water % (v/v).

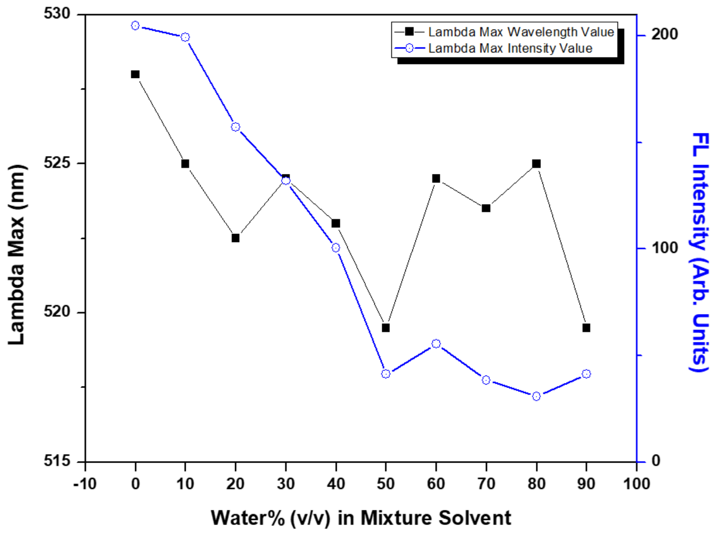

Figure S14. Fluorescence intensity and emission peak of polymer **5** as a function of water content in CH_3OH (1µM repeating units, λ_{ex} at 336 nm).
